# General-Purpose Artificial Intelligence Use in Routine Mental Health Practice Among Australian Clinicians: Mixed Methods Study

**DOI:** 10.2196/101629

**Published:** 2026-08-31

**Authors:** Benjamin Johnson, Tingting Yang, Daniel Stjepanović, Tianze Sun, Gary Chan, Janni Leung

**Affiliations:** 1 National Centre for Youth Substance Use Research, School of Psychology, The University of Queensland, 31 Upland Road, Brisbane, Queensland, 4067, Australia, 61 7 3343 252; 2 Domestic Violence Action Centre, Ipswich, Queensland, Australia

**Keywords:** artificial intelligence, large language models, mental health, clinicians, psychotherapy, digital mental health, mixed methods, clinical governance

## Abstract

**Background:**

General-purpose AI tools are increasingly accessible to mental health professionals, yet little is known about how these off-the-shelf systems are being integrated into routine therapeutic practice.

**Objective:**

This study aimed to examine how Australian mental health professionals are using general-purpose AI tools in routine practice, including patterns of use, perceived usefulness, concerns, workplace governance, and factors associated with frequent AI use.

**Methods:**

We conducted a sequential mixed methods study with Australian mental health professionals. Semistructured qualitative interviews were conducted with 12 clinicians recruited through the research team’s professional networks between June 1, 2025, and August 10, 2025, to explore current uses, perceived benefits, limitations, and governance issues. Interview data were analyzed using deductive qualitative content analysis, guided by predefined domains from the interview guide. Findings from the qualitative phase informed a national survey of 278 respondents. Survey respondents were recruited using convenience sampling through physical posters, university networks, social media, organizations, Primary Health Networks, newsletters, and direct contact with psychology and counseling clinics. Survey recruitment occurred from September 1, 2025, to April 1, 2026. Survey analyses included descriptive statistics, bivariate tests, and multivariable logistic regression models examining daily AI use across client-facing and administrative or clinician-support tasks, perceived performance, concerns of use, workplace permissions, and demographic and professional factors associated with daily use.

**Results:**

Interview participants described AI use across client-facing, administrative, documentation, translation, planning, research, and emotional-support tasks. Qualitative findings indicated that clinicians used AI primarily as a clinician-supervised support tool, particularly for saving time, reducing cognitive load, supporting documentation, and improving in-session focus. Participants also raised concerns about privacy, data security, accuracy, client acceptability, overreliance, loss of human judgment, and uneven workplace governance. Survey findings showed that 121 of 278 respondents (43.5%) reported daily administrative or clinician-support AI use, and 92 of 278 respondents (33.1%) reported daily client-facing AI use. Commonly endorsed concerns included privacy and security, accuracy, insufficient training or support, limited confidence in judging outputs, and a preference for human judgment. In adjusted cross-sectional analyses, speaking a language other than English at home was statistically associated with daily administrative or clinician-support AI use and daily client-facing AI use. Age, years practicing, gender, education, and profession were not independently associated with either outcome.

**Conclusions:**

General-purpose AI tools appear to be entering routine mental health practice across a broad range of tasks, particularly as clinician-supervised workflow support tools. However, uptake is occurring alongside unresolved concerns about privacy, accuracy, training, human oversight, and workplace governance. Clearer guidance, practical training, and task-specific evaluation are needed to support safe and appropriate AI integration into mental health care.

## Introduction

Mental health systems are under growing strain worldwide. According to the World Health Organization, more than 1 billion people are living with mental health conditions, and services require urgent scale-up [1]. In some countries, up to 90% of people with severe mental health conditions receive no care at all, underscoring the scale of unmet need and the limits of existing service capacity. Against this backdrop, interest has grown in AI tools that may support mental health care delivery as scalable adjuncts to care [2].

AI has been proposed to support or deliver therapeutic interventions in mental health, including direct client interaction and clinician-guided applications that augment care [3]. These applications may include direct client interaction [3-6] and clinician-support functions such as documentation [7], treatment planning [3-5,7], psychoeducation [5,7], and research [4].

Existing research on AI in mental health has focused largely on chatbots and their use in direct interaction with users [6,8]. Less attention has been given to general-purpose, off-the-shelf AI tools that clinicians may use to support or augment routine therapeutic practice [9]. In Australia, a survey of mental health professionals found that 43% had used AI in the previous 6 months, primarily for research and report or letter writing [4]. However, detailed evidence on how these tools are integrated across routine therapeutic tasks and governed within Australian workplaces remains limited. This is an important gap because these tools are among the most widely accessible forms of AI and may hold particular potential for extending mental health support in low-resource settings where access to specialist tools and services is limited [10].

Accordingly, the value of these tools depends not only on their performance, but also on how they are used by clinicians and implemented in routine practice. Understanding how mental health professionals are already using these tools; how useful they perceive them to be across different tasks; and what concerns, risks, and workplace conditions shape their use is therefore important for understanding how AI is being integrated into real-world therapeutic practice.

Therefore, this study examined how general-purpose, off-the-shelf AI tools are being integrated into routine therapeutic practice by mental health professionals. The aims were to examine patterns of use across client-facing and clinician-support tasks; assess perceived usefulness across these applications; identify key limitations, risks, and ethical concerns; and explore the individual and workplace factors associated with AI use, including governance, permissions, and training.

## Methods

### Study Design and Participants

This study used a sequential mixed-methods design. In this study, we investigated the use of general-purpose AI, which referred to publicly accessible or commercially available AI tools that clinicians could use across multiple routine practice tasks, rather than tools developed specifically for this study or disorder-specific digital mental health interventions. This included large language model (LLM) chatbots, AI-enabled documentation or transcription tools, translation tools, and other off-the-shelf AI systems used for administrative, clinician-support, or client-facing tasks.

The qualitative phase captured participant-described use of tool categories, including LLM chatbots, AI-enabled clinical scribes and documentation systems, transcription tools, and translation tools. The quantitative survey assessed AI use across specified clinical and clinician-support tasks rather than asking respondents to identify the specific platform or tool category used.

First, semistructured qualitative interviews were conducted with Australian mental health professionals to explore how AI, including LLMs, was being used in therapeutic practice, how clinicians perceived its accuracy and reliability, and what factors shaped openness or reluctance toward its use. Findings from the qualitative phase were then used to inform the development of a national quantitative survey examining patterns of AI use, perceived effectiveness, concerns, and workplace governance among mental health professionals.

### Ethical Considerations

Ethics approval was obtained from The University of Queensland Human Research Ethics Committee (2025/HE000535). All participants provided informed consent before participating. For the qualitative phase, participants provided consent before the interview, including consent for the interview to be audio-recorded and transcribed. For the quantitative phase, participants provided consent electronically before commencing the Qualtrics survey.

Participant privacy and confidentiality were protected by deidentifying data used for analysis and reporting. Interview transcripts were not made publicly available because they may contain information that could compromise participant privacy. Deidentified quantitative data may be made available by the corresponding author upon reasonable request, subject to ethics approval and participant consent conditions.

### Qualitative Phase

#### Recruitment and Data Collection

Participants for the qualitative phase were recruited through the research team’s local professional networks by contacting psychologists and counselors within those networks. Recruitment was convenience-based, with eligible clinicians contacted based on accessibility through the research team’s networks and willingness to participate. Participants were not selected according to prespecified quotas for profession, workplace setting, AI use status, or demographic characteristics. Both clinicians who used AI and those who did not use AI were eligible to participate. Recruitment for the qualitative phase occurred between June 1, 2025, and August 10, 2025. Interviews were conducted during the same period.

Interviews were conducted by BJ, a PhD candidate with training in psychology, addiction research, qualitative research, and mixed-methods research, and TY, a practicing counselor with training in psychology and counseling. Both researchers had an interest in understanding how AI was being used in therapeutic practice, and some participants had prior professional relationships with members of the research team because recruitment occurred through professional networks.

Participants received Aus $20 (Aus $1=US $0.6528 as of August 8, 2025) for their participation. Recruitment continued until pragmatic category saturation was judged to have been reached. Saturation was assessed during data collection through ongoing review and discussion of interview transcripts and coding summaries by BJ and TY. Recruitment was stopped when additional interviews were no longer identifying new major categories relevant to the study aims, including AI use cases, perceived benefits, limitations, privacy and accuracy concerns, workplace governance, and training needs. Given the deductive content analysis approach, saturation was interpreted as sufficient coverage of the predefined interview domains and recurring categories needed to inform survey item development, rather than as theoretical saturation. The semistructured interviews were conducted with 12 Australian mental health professionals by 2 authors (BJ and TY) on Zoom (Zoom Communications, Inc). Interviews were audio-recorded and transcribed using Otter.ai (Otter.ai Inc). The interview guide focused on four areas: (1) how participants used AI in their work, (2) their perceptions of the accuracy and reliability of AI tools, (3) factors influencing openness or reluctance toward AI use in therapy, and (4) workplace policy and AI integration. BJ and TY asked follow-up or probing questions where clarification or elaboration was needed, and did not repeat questions when the relevant topic had already been addressed in an earlier response. The full interview guide is provided in Multimedia Appendix 1.

#### Qualitative Analysis and Survey Development

Interview data were analyzed using deductive qualitative content analysis [11]. The overarching analytic structure was informed by four predefined domains from the interview guide: (1) how participants used AI in their work, (2) perceptions of its accuracy and reliability, (3) factors influencing openness or reluctance toward its use, and (4) workplace policy and AI integration. Within this structure, recurring categories and codes were identified and refined from the transcripts, informing the 3 reported themes and subsequent survey-item development.

Coding was conducted by BJ and TY using NVivo 14 (QSR International). After familiarizing themselves with the data, both researchers independently coded all interview transcripts and developed initial code lists. During weekly meetings, they compared code labels, definitions, and their application to specific excerpts; reviewed differences against the source transcripts; and revised the coding framework through consensus. Both researchers then recoded all transcripts using the agreed framework, which was refined iteratively as categories were clarified, combined, or separated. Differences in interpretation were resolved through discussion. GC, DS, JL, and TS subsequently reviewed the emerging categories and themes, provided feedback on their interpretation and organization, and reviewed and approved the final themes. Formal intercoder reliability statistics were not calculated because agreement was established through this iterative consensus process.

Following coding, BJ and TY reviewed the coded excerpts and coding summaries to identify categories relevant to survey development. These categories were organized into survey areas covering AI-use tasks, perceived benefits, concerns and risks, workplace permissions, training needs, and governance, and were translated into draft survey items. For example, AI-assisted note-taking, transcription, translation, treatment planning, research, client education, and emotional support informed task-frequency items, while privacy, accuracy, confidence in judging outputs, client acceptability, overreliance, and preference for human judgment informed concern items. Relevant literature on AI use in mental health care and clinical documentation was used to check item coverage, and the items were refined through author-team review to improve clarity and alignment with the study aims. The final survey contained 16 substantive questions, comprising 89 potential response items when individual matrix rows and multiple-response options were counted separately. The number of items displayed varied between respondents because follow-up items assessing effectiveness and performance relative to humans were shown only for AI tasks that respondents reported having used. The qualitative component was reported in accordance with the Consolidated Criteria for Reporting Qualitative Research (COREQ) guidelines, with the completed checklist provided in Checklist 1.

Member checking was not undertaken. Transcripts were not returned to participants, and participants were not invited to comment on the findings. Credibility was supported through independent coding by 2 researchers, consensus discussions, wider-team review of categories and themes, and the presentation of participant quotations alongside the findings.

The developmental integration between the qualitative and quantitative phases is presented in Table S1 in Multimedia Appendix 1, which maps the qualitative themes and interview-derived categories to the corresponding quantitative survey questions and items.

Formal pilot testing with external clinicians was not conducted. The draft survey was pretested by all coauthors before recruitment commenced. Each coauthor completed the survey once to check item clarity, survey flow, skip logic, and potential errors.

### Quantitative Phase

#### Recruitment

Participants for the survey were recruited using convenience sampling. Recruitment methods included physical posters displayed at universities in Brisbane and Sydney; distribution through university networks across Australia; promotion through social media, including LinkedIn; circulation through organizations, Primary Health Networks, and newsletters; and direct contact with psychology and counseling clinics. The survey was hosted on Qualtrics, and participants provided consent before commencing the survey. Participants who completed the survey could opt into a raffle draw, in which 1 of every 5 entrants won a Aus $50 multipurpose electronic gift card. Recruitment commenced on September 1, 2025, and continued until April 1, 2026.

#### Measures

The survey assessed demographic and professional characteristics, including age, gender, education, years practicing therapy, profession, language spoken at home, and workplace type.

We assessed the frequency of AI use across 12 tasks in the past 8 weeks: chatting directly with a client, transcribing sessions, preparing administrative documents, treatment planning, client education, research and information finding, practicing skills, language translation, screening or triage questionnaires, formal diagnosis, informal supervision or guidance, and personal emotional support.

We then assessed the perceived effectiveness of AI for each task among respondents with personal experience using AI for that task, operationalized on a 5-point Likert scale ranging from much worse to much better. Comparative judgments of AI relative to humans were assessed for each task. Reasons for AI use, concerns about AI use, workplace permissions for AI use in administrative or documentation tasks and during client sessions, and whether respondents had clear written workplace AI policies or guidelines were also assessed.

For analyses examining workplace regulations, workplace permission responses were collapsed into 4 ordered categories: banned, unclear, allowed at clinician discretion, and encouraged/required. The encouraged and required categories were combined because of small cell sizes.

Two binary outcomes were created to capture frequent AI use: daily use for client-facing tasks and daily use for administrative or clinician-support tasks. The client-facing outcome was based on 3 items: chatting directly with clients, transcribing sessions, and language translation. The administrative or clinician-support outcome was based on 9 items: preparing administrative documents, treatment planning, client education, research and information seeking, practicing skills, screening or triage questionnaires, formal diagnosis, informal supervision or guidance, and personal emotional support.

Participants were coded positive if they reported daily AI use on at least one task in the relevant category. All items were originally measured on a 5-point scale: never, rarely, monthly, weekly, and daily, and were then dichotomized into daily versus less than daily use. Daily use was selected as the threshold for frequent AI use because broader thresholds, such as weekly or more, classified a very high proportion of respondents as frequent users when multiple tasks were combined, limiting variability and reducing the usefulness of regression modeling. The daily threshold therefore provided a more discriminating indicator of frequent use.

Age and years practicing therapy were entered as continuous variables. For the regression analyses, responses to the gender item were limited to the male and female response categories because there were too few respondents in the other categories to support stable estimation. Language spoken at home was coded as English only versus language other than English, with “prefer not to say” excluded. Education was dichotomized as master’s degree or doctorate versus all other education levels. This recoding was adopted because the available education variable combined bachelor and graduate diploma or certificate qualifications, which precluded a cleaner postgraduate versus nonpostgraduate classification. Profession was recoded into 6 categories: psychologist, counselor, social worker, occupational therapist, psychiatrist, and other. The psychologist category included provisional psychologists, and the other category included mental health nurses, peer workers, and other professions. Psychologist was used as the reference group for profession, and all other education levels were used as the reference group for education.

#### Bivariate Analyses

Quantitative analyses were conducted in R version 4.5.3 (R Project for Statistical Computing). Responses were excluded if participants completed the survey in under 3 minutes or did not complete the survey.

Descriptive comparison tables were generated for the 2 daily AI-use outcomes: daily client-facing AI use and daily administrative or clinician-support AI use. These tables reported overall means, SDs, and ranges for continuous variables, and frequencies and percentages for categorical variables. Within each category, the number and percentage of respondents classified as daily AI users and not daily AI users were also reported. For bivariate comparisons, independent-samples *t* tests were used to examine associations between continuous variables and each of the 2 daily AI-use outcomes, and Pearson chi-square tests were used to examine associations between categorical variables and each of the 2 daily AI-use outcomes. Categories with very small cell sizes were excluded from inferential testing.

To examine cross-sectional associations between endorsed concerns and daily AI use, each concern item was analyzed separately as a binary indicator of whether the respondent had endorsed it. Because multiple concern items were examined, *P* values were adjusted using the Benjamini-Hochberg false discovery rate procedure, applied separately within each outcome model set. This approach controls the expected proportion of false discoveries among statistically significant findings while maintaining greater power than more conservative family-wise error corrections [12].

#### Multivariable Regression Analyses

Multivariable logistic regression models were fitted to examine associations between respondent characteristics and frequent AI use. Separate models were estimated for daily client-facing AI use and daily administrative or clinician-support AI use. Predictors included age, years practicing therapy, gender, language other than English spoken at home, education, and profession.

Workplace type was not included in the multivariable models because it was measured as a multiple-response item across 12 nonmutually exclusive settings, and no primary workplace was identified. Including all settings as separate indicators would have substantially increased the number of model parameters and introduced sparse cells, whereas collapsing them would have obscured meaningful differences between workplace contexts.

Missing data in the multivariable regression models resulted from analytic recoding decisions rather than item nonresponse. Eight respondents who selected other response categories for the gender item were excluded because the cell counts were insufficient for stable modeling, and 1 respondent who selected “prefer not to say” for language spoken at home was excluded. Consequently, both multivariable models included 269 respondents. No imputation was performed.

As language translation was one of the tasks included in the client-facing daily AI use outcome, and language other than English spoken at home emerged as a significant correlate of client-facing AI use, a post hoc sensitivity analysis was conducted. In this analysis, the client-facing outcome was redefined to exclude language translation, and the multivariable logistic regression model was rerun to assess whether the observed association remained.

### Mixed-Methods Integration

After the qualitative and quantitative analyses were completed, findings from both phases were compared during interpretation. Qualitative categories were matched with related survey domains and quantitative results to assess whether the survey findings supported, extended, or differed from the interview findings. This comparison was presented in a joint display and informed the overall interpretation of the study findings.

## Results

### Overview

The study comprised qualitative interviews with 12 Australian mental health professionals and a quantitative survey of 278 respondents. The qualitative findings are presented first because they informed the development and organization of the survey items, and they are followed by the quantitative results describing patterns of AI use, perceived performance, concerns, workplace permissions, and factors associated with daily AI use.

### Qualitative Findings

#### Overview

The 12 mental health professionals practicing in Australia who were interviewed spanned a range of professional roles, practice settings, training backgrounds, and experience levels (Table 1).

The qualitative analysis generated 3 themes and 8 subthemes, which are summarized in Table 2.

**Table 1.** Sample characteristics of qualitative interview participants (n=12).

Participant	Professional role	Practice setting	Training background	Years practicing
P1	Psychologist/provisional psychologist	Private practice	Psychology; counseling	0‐2
P2	Counselor	School, education, or training clinic	Psychology; counseling	2‐5
P3	Psychologist/provisional psychologist	School, education, or training clinic	Psychology	0‐2
P4	Counselor	School, education, or training clinic	Psychology	5‐10
P5	Counselor	Community, crisis, health, or multidisciplinary	Psychology; counseling; social work	>10
P6	Support worker	Community, crisis, health, or multidisciplinary	Psychology; social work	0‐2
P7	Psychologist/provisional psychologist	Community, crisis, health, or multidisciplinary	Psychology	2‐5
P8	Counselor	Community, crisis, health, or multidisciplinary	Counseling; occupational therapy	2‐5
P9	Counselor	Community, crisis, health, or multidisciplinary	Counseling	2‐5
P10	Psychologist/provisional psychologist	Community, crisis, health, or multidisciplinary	Psychology	>10
P11	Social worker	Community, crisis, health, or multidisciplinary	Social work	2‐5
P12	Counselor	School, education, or training clinic	Psychology; counseling	2‐5

**Table 2.** Current functions and perceived effectiveness.

Theme and subtheme		Codes^a^
Theme 1: Current functions and perceived effectiveness (n=12)		
1.1 In-session use (n=8)		AI-assisted note-taking, transcription, and case-note generation (n=6) Reduced cognitive load and preserved decompression time (n=3) Improved focus and therapeutic engagement during sessions (n=2) Translation when an interpreter is unavailable (n=1)
1.2 Out-of-session use (n=10)		Administrative drafting outside sessions, including letters, reports, emails, and referrals (n=4) Clinical brainstorming and treatment planning support (n=7) Psychoeducation or workshop materials (n=5) Unfamiliar-topic learning/early stage research orientation (n=4) Provisional diagnostic prompting/idea generation (n=1)
1.3 Direct psychological support (n=6)		Emotional validation/reflection for clinicians (n=4) Client coping support between sessions (n=3) Accessibility and 24/7 support (n=3)
Theme 2: Present limitations and reasons for nonuse (n=12)		
2.1 Accuracy and reliability concerns (n=9)		Hallucinated references (n=3) Misleading or incomplete information (n=5) Overgeneral responses (n=2) Inaccurate transcripts or missed key session details (n=4) Need for active checking and verification (n=7)
2.2 Privacy, security, and client acceptability (n=10)		Confidentiality concerns (n=8) Uncertainty about data storage (n=4) Client discomfort and nonconsent (n=3)
2.3 The human touch (n=8)		AI can simulate empathy but not genuine attunement (n=4) Inability to read body language or nonverbal cues (n=4) Limits in responding flexibly in emotionally complex situations (n=5) Therapeutic relationship as fundamentally human (n=6)
Theme 3: Integration into practice (n=12)		
3.1 Future projections of AI in mental health therapy (n=12)		Supplement, not replacement (n=9) Partial replacement of discrete structured tasks (n=3) Concern uptake may be driven by corporate or economic pressures (n=1)
3.2 Influence of workplace policy and training (n=10)		Uneven workplace governance/patchwork rules (n=8) Explicit bans or substitution of one tool for another (n=2) Desire for training and upskilling of AI skills (n=5) Need for practical guidance on safe, effective, context-sensitive use (n=6)

a Codes reported by 1 participant (n=1) are presented as illustrative examples of variation within the broader subtheme and have not been treated as standalone themes.

#### Theme 1: Current Functions and Perceived Effectiveness

##### In-Session Use

Participants described using AI during or immediately around live client contact, primarily to support documentation while improving in-session presence. The most common use involved tools, such as Heidi Health, Halaxy, and Nova Note, to record sessions, generate transcripts, and assist with case note preparation.

Participants described this use as reducing the need to mentally track details, allowing greater focus on the client and therapeutic engagement. Participant P12 explained as follows:

*...for case note…that helps you better focusing during the session... you get no longer distracted by trying to remember all the things... it improves the quality of your therapeutic engagement...” Some also described reduced cognitive load and more time to decompress after demanding sessions. As P9 noted: “Clinicians are still human beings at the end of the day, and we do need those 30 minutes to decompress… the pressure to complete the case notes eats away that time when you’re supposed to relax.*

In addition to documentation support, participant P6 described using AI for translation when working with clients who spoke different languages, particularly when an interpreter was unavailable. Participant P6 also viewed this as more effective than conventional translation tools and made the following statement:

*...one thing I usually use... with ChatGPT, I use it for translation, and I think that one is more accurate than Google Translator a lot of the time...*

##### Out-of-Session Use

Participants described using AI for a wide range of purposes outside of live client contact. Most commonly, this involved improving efficiency in administrative and documentation tasks, including drafting letters to general practitioners, reports, emails, and referrals. Participants referred to using tools, such as Gemini, NovoNote, and ChatGPT, for these purposes, positioning AI as a practical aid for managing routine written work outside sessions.

Participants also described using AI to support planning and research related to client care, particularly when working with complex presentations or feeling stuck on a case. In these instances, AI was used as a starting point for generating ideas rather than as a standalone source of authority. Participant P6 explained as follows:

*Some of my clients has really complex mental health issues... so I’ll just put it into chat GPT, and see what it comes up with.*

Participant P7 described a similar use as follows:

*...there were a few times where it kind of came up with, like, provisional diagnosis... sometimes I’d be like, yeah, I was kind of thinking that. And then sometimes I’d be like, oh, wow. I didn’t consider that at all...*

Some participants also used AI to learn about unfamiliar topics. Participant P10 explained as follows:

*...I sometimes tap into ChatGPT when I’m starting to do a little bit of research, or if there’s like an area that I’m not very familiar with, for example if I want to learn CBT ask for it to search articles researchers or prominent people in the field who are experts to learn more about it...*

##### Direct Psychological Support

Participants also described AI as an immediate source of reflection, validation, and coping support outside formal sessions for both clinicians and clients. Several participants described turning to ChatGPT during periods of stress, frustration, or burnout. Participant P9 explained as follows:

*...during a period of time, I felt especially burned out... I just sensed my own frustration, and I just dumped my shit [into ChatGPT], and it validated me. It’s really good.*

Participants also described clients using ChatGPT in this way, particularly as a coping tool outside therapy sessions. Participant P2 noted that this was especially common among their younger clients:

*Some students... will literally... talk with chat GPT as their coping strategy... this person has no trusted adult or no trust person around them and ChatGPT is kind of the last resort...*

Accessibility was central to why participants saw AI as useful in this context, offering immediate support at moments of need, including between appointments or late at night. Participant P10 commented as follows:

*I’m not going to be with my clients, 24/7 but the AI technology is on their phone, and so they could have the additional support while seeing me...*

### Theme 2: Present Limitations and Reasons for Nonuse

#### Accuracy and Reliability Concerns

Participants commonly raised concerns about the accuracy and reliability of AI outputs, warning that these tools could produce mistakes, misleading information, or incomplete accounts. Participant P4 described this as a problem inherent to how LLMs generate responses:

*...with AI, as I understand it, it just it draws its knowledge kind of like a huge vacuum, and it takes in all the good and the bad... there’s a lot of... half truths out there.*

Participant P4 added that “...a little knowledge can be a dangerous thing....”

These concerns also extended to out-of-session uses such as literature searching or idea generation. Participants noted that AI could produce inaccurate references or attribute information to the wrong sources, making it difficult to trust without verification. For this reason, several participants suggested that AI outputs could not be taken at face value and instead required active checking and monitoring by the user. Participant P11 stated:

*...you’ve got to be really careful with AI... you’ve got to be a smart person to use AI... you can’t just be someone going into it without an idea of what you’re using...*

Participants also described similar limitations in in-session uses such as transcription and case note generation. Participants noted that AI-generated notes could add, omit, or distort important session details, meaning that outputs still required careful review and editing before use.

#### Privacy, Security, and Client Acceptability

Participants also raised concerns about privacy, security, and client acceptability, particularly when using general-purpose tools such as ChatGPT. A key concern was whether sensitive client information could be collected, stored, or used in ways not fully understood by clinicians or clients.

Several participants were uncertain about what happens to information once it is entered into an LLM, contributing to discomfort about using these tools in therapeutic contexts. Participant P10 explained as follows:

*...what is interesting for me is the privacy aspects of things. So who owns the data when it goes into the large language models...*

Participants noted that these concerns were often shared by clients, with some not consenting to in-session AI use because of privacy concerns.

#### The Human Touch

Participants raised concerns that although AI can generate empathy-like responses based on learned linguistic patterns, it lacks the presence, attunement, affective empathy, genuine emotional understanding, and relational nuance needed for therapeutic work. Therapy was described as a distinctly human process that depended on reading body language, responding flexibly, and validating the person in front of you. Participant P1 explained that there is an “...art to therapy...you ask certain questions based on picking up body language, body cues....”

Participants also framed this limitation in terms of client preferences, suggesting that many clients still wanted to speak to another person, particularly in emotionally demanding or complex situations.

Participant P4 also questioned whether AI could reproduce the therapeutic relationship, asking the following question:

*Can a person have a therapeutic relationship with a program?*

They further questioned whether simulated empathy would be sufficient and suggested that more difficult interpersonal work, such as building rapport and appropriately challenging clients, would be difficult to replicate without that relationship.

### Theme 3: Integration Into Practice

#### Future Projections of AI in Mental Health Therapy

Participants generally viewed AI as having a place in therapy-related work, but primarily as a supplement rather than a replacement for clinicians. Participant P8 described AI as a “personal assistant” and emphasized the need for clinician discretion as follows:

*We need to have our own insight and judgment to think for ourselves. We can’t just make ChatGPT words as gospel.*

Some participants nevertheless saw scope for partial replacement in narrower areas of practice, particularly where work was more structured, skills-based, or educational in nature. Participant P2 noted that AI may be especially suited to support that does not depend on the full depth of a therapeutic relationship, including strategy- or solution-focused input available “...24/7, something that human therapists could not offer....”

Participant P5 suggested that future integration may be shaped less by clinical usefulness than by broader economic and industry pressures. Moreover, participant P5 argued that “...corporations are already driving the industry...,” raising concern that adoption may expand without sufficient evidence or oversight.

#### Influence of Workplace Policy and Training

Participants described AI integration as shaped by workplace policies, governance, and organizational expectations as well as individual choice. These arrangements were uneven across settings. In some workplaces, access to particular tools had been explicitly restricted because of security concerns. Participant P7 stated:

*...we’ve banned chat GPT, but we’re using copilot instead, which is very inferior.*

In other settings, participants described uncertainty about what was and was not permitted. Some reported that AI use was restricted despite the absence of clear written guidance, while others described AI being used informally or privately without explicit organizational direction. For example, participant P12 noted:

*...it’s not clearly saying you are encouraged to use that. But I know lots of people are... using that like privately...*

Participants in private practice described a different context, where the absence of formal governance meant responsibility sat more heavily with the individual practitioner.

Participants also expressed a need for more training to support appropriate, safe, and context-sensitive AI use. Participant P2 noted that using AI sensitively was becoming “...more of a skill...” that “...needs to be taught...,” while participant P3 mentioned that existing training focused more on security than on how to use AI effectively and safely in practice.

The qualitative findings indicated that AI use in therapeutic practice occurred across several domains, including in-session documentation support, out-of-session administrative and clinical tasks, translation, research, planning, and emotional support. They also identified key concerns shaping use, including privacy and security, accuracy, client acceptability, preference for human judgment, workplace governance, and training. These categories informed the development and organization of the quantitative survey items, including task-frequency items, perceived performance ratings, self-reported reasons for use, concern items, and workplace permission measures examined in the quantitative phase.

### Quantitative Findings

#### Sample Characteristics and Bivariate Associations

The quantitative descriptive sample included 278 respondents. Of these, 121 (43.5%) reported daily administrative or clinician-support AI use and 92 (33.1%) reported daily client-facing AI use. The mean age was 35.60 years, and respondents had been practicing therapy for a mean of 7.12 years. Among the respondents, 58.1% (157/270) were female, 54.7% (152/278) had completed a master’s degree or doctorate, and 43.3% (120/277) reported speaking a language other than English at home (Table 3).

**Table 3.** Participant characteristics by daily administrative or clinician-support AI use and daily client-facing AI use (N=278).

Variable		Overall	Daily use	Less than daily use	Chi-square (*df*)	*t* test (*df*)	*P* value
Administrative or clinician-support AI use							
Age (years)					—^a^	0.10 (274.3)	.92
Mean (SD)		35.60 (9.11)	35.55 (8.17)	35.65 (9.79)			
Range		20.0-67.0	—	—			
Years practicing therapy					—	−0.75 (276.0)	.45
Mean (SD)		7.12 (6.05)	7.43 (5.14)	6.89 (6.67)			
Range		0.1-37.0	—	—			
Gender, n (%)					1.57 (1)	—	.21
Female		157 (58.1)	63 (40.1)	94 (59.9)			
Male		113 (41.9)	54 (47.8)	59 (52.2)			
Language other than English at home, n (%)					10.14 (1)	—	.001
No		157 (56.7)	55 (35.0)	102 (65.0)			
Yes		120 (43.3)	65 (54.2)	55 (45.8)			
Highest education completed, n (%)					0.48 (1)	—	.49
All other		126 (45.3)	52 (41.3)	74 (58.7)			
Master’s degree or doctorate		152 (54.7)	69 (45.4)	83 (54.6)			
Current profession, n (%)					9.11 (5)	—	.11
Psychologist		84 (30.2)	34 (40.5)	50 (59.5)			
Counselor		67 (24.1)	24 (35.8)	43 (64.2)			
Social worker		37 (13.3)	16 (43.2)	21 (56.8)			
Occupational therapist		32 (11.5)	19 (59.4)	13 (40.6)			
Psychiatrist		26 (9.4)	16 (61.5)	10 (38.5)			
Other		32 (11.5)	12 (37.5)	20 (62.5)			
Client-facing AI use							
Age (years)					—	−1.52 (167.3)	.13
Mean (SD)		35.60 (9.11)	36.82 (9.65)	35.01 (8.79)			
Range		20.0-67.0	—	—
Years practicing therapy					—	−2.14 (149.7)	.03
Mean (SD)		7.12 (6.05)	8.31 (6.91)	6.54 (5.50)			
Range		0.1-37.0	—	—
Gender, n (%)					0.19 (1)	—	.66
Female		157 (58.1)	54 (34.4)	103 (65.6)			
Male		113 (41.9)	36 (31.9)	77 (68.1)			
Language other than English at home, n (%)					8.23 (1)	—	.004
No		157 (56.7)	41 (26.1)	116 (73.9)			
Yes		120 (43.3)	51 (42.5)	69 (57.5)			
Highest education completed, n (%)					2.94 (1)	—	.09
All other		126 (45.3)	35 (27.8)	91 (72.2)			
Master’s degree or doctorate		152 (54.7)	57 (37.5)	95 (62.5)			
Current profession, n (%)					5.04 (5)	—	.41
Psychologist		84 (30.2)	29 (34.5)	55 (65.5)			
Counselor		67 (24.1)	20 (29.9)	47 (70.1)			
Social worker		37 (13.3)	11 (29.7)	26 (70.3)			
Occupational therapist		32 (11.5)	14 (43.8)	18 (56.2)			
Psychiatrist		26 (9.4)	11 (42.3)	15 (57.7)			
Other		32 (11.5)	7 (21.9)	25 (78.1)			

a Not applicable.

#### Categories With Small Cell Counts Were Excluded

Bivariate comparisons by daily AI use are shown in Table S2 in Multimedia Appendix 1. Age was not associated with either outcome. Years practicing therapy was not associated with daily administrative or clinician-support AI use, but respondents reporting daily client-facing AI use had practiced for longer on average than those who did not (*t*_149.7_=−2.14). Among respondents who spoke a language other than English at home, 54.2% (65/120) reported daily administrative or clinician-support AI use and 35.0% (55/157) did not (*χ*²₁=10.14). Similarly, among this group of respondents, 42.5% (51/120) reported daily client-facing AI use and 26.1% (41/157) did not (*χ*²₁=8.23). Gender, education, and profession were not associated with either outcome.

#### Patterns of AI Use Across Tasks

Patterns of AI use across therapeutic and clinician-support tasks over the past 8 weeks are shown in Table 4. Daily use frequencies for each task are also presented in Table S3 in Multimedia Appendix 1. The highest levels of daily use were reported for research and information finding (59/278, 21.2%), language translation (51/278, 18.3%), and transcribing sessions (49/278, 17.6%). The lowest levels of daily use were reported for formal diagnosis (14/278, 5.0%), treatment planning (18/278, 6.5%), and screening or triage questionnaires (19/278, 6.8%).

**Table 4.** Frequency of AI use across therapeutic and clinician-support tasks in the past 8 weeks.

Task	Less than weekly (N=278), n (%)	Weekly (N=278), n (%)	Daily (N=278), n (%)
Research and information finding	107 (38.5)	112 (40.3)	59 (21.2)
Language translation	174 (62.6)	53 (19.1)	51 (18.3)
Transcribe sessions	137 (49.3)	92 (33.1)	49 (17.6)
Own emotional support	176 (63.3)	57 (20.5)	45 (16.2)
Prepare administrative documents	147 (52.9)	95 (34.2)	36 (12.9)
Chat directly with a client	204 (73.4)	48 (17.3)	26 (9.4)
Client education	182 (65.5)	70 (25.2)	26 (9.4)
Practice skills	196 (70.5)	58 (20.9)	24 (8.6)
Screening or triage questionnaires	215 (77.3)	44 (15.8)	19 (6.8)
Informal supervision or guidance	198 (71.2)	61 (21.9)	19 (6.8)
Treatment planning	198 (71.2)	62 (22.3)	18 (6.5)
Formal diagnosis	223 (80.2)	41 (14.7)	14 (5.0)

#### Perceived Performance of AI Relative to Humans

**Figure 1.**
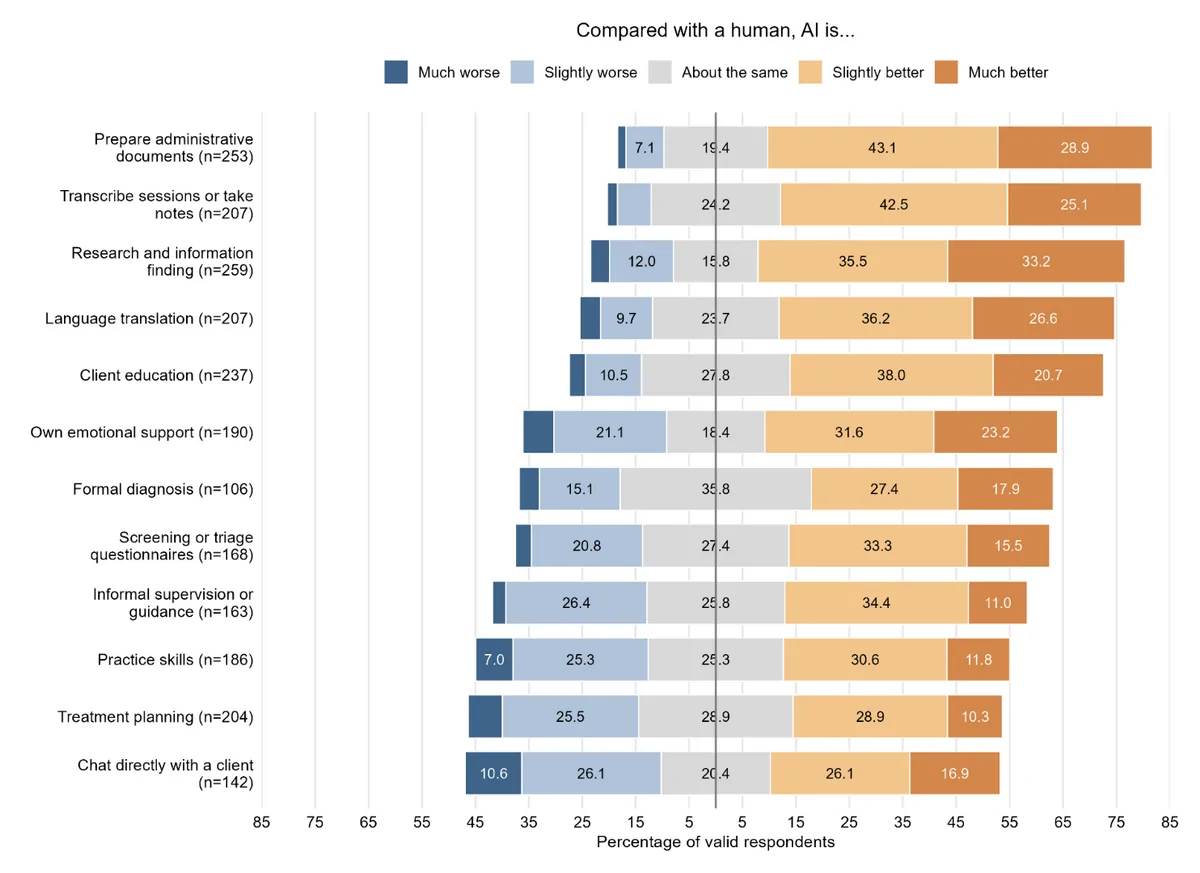
Perceived performance of AI relative to humans across therapeutic and clinician-support tasks.

Perceived performance ratings among respondents with experience using AI for each task are shown in Figure 1, with complete distributions and per-task denominators presented in Table S4 in Multimedia Appendix 1. Combining responses of *slightly better* and *much better*, the highest favorable rating was for preparing administrative documents: 182 of 253 respondents (71.9%, 95% CI 66.1%‐77.1%) rated AI as better than a human, compared with 49 (19.4%) who rated it about the same and 22 (8.7%) who rated it worse. Favorable ratings were also common for research and information finding (178/259, 68.7%, 95% CI 62.8%‐74.1%), transcription or note-taking (140/207, 67.6%, 95% CI 61.0%‐73.6%), language translation (130/207, 62.8%, 95% CI 56.0%‐69.1%), client education (139/237, 58.6%, 95% CI 52.3%‐64.7%), and clinicians’ own emotional support (104/190, 54.7%, 95% CI 47.6%‐61.7%). Fewer than half of respondents rated AI as better than a human for screening or triage questionnaires (82/168, 48.8%), informal supervision or guidance (74/163, 45.4%), formal diagnosis (48/106, 45.3%), chatting directly with a client (61/142, 43.0%), practicing skills (79/186, 42.5%), or treatment planning (80/204, 39.2%). For direct client interaction, 29 of 142 respondents (20.4%) rated AI as about the same and 52 (36.6%) rated it as worse than a human. For treatment planning, 59 of 204 respondents (28.9%) rated it as about the same and 65 (31.9%) rated it as worse.

#### Reasons for Use and Concerns About Use

Self-reported reasons for using AI are shown in Figure S1 in Multimedia Appendix 1, and endorsed concerns about AI use are shown in Figure S2 in Multimedia Appendix 1. The most endorsed reason was that AI saves time and improves productivity (216/278, 77.7%), followed by perceived benefits for work quality, cognitive load, and in-session presence. The most endorsed concerns were privacy and security concerns (175/278, 62.9%) and concerns about accuracy (158/278, 56.8%), followed by preferring human judgment and concerns about overreliance, client discomfort, and insufficient training or support.

#### Concerns and Daily AI Use

Associations between endorsed concerns and daily AI use are shown in Table 5. For daily administrative or clinician-support AI use, preferring human judgment was the only concern associated with lower odds of use after false discovery rate correction (odds ratio [OR] 0.39, 95% CI 0.21-0.73). For daily client-facing AI use, lower odds were observed for privacy/security concerns (OR 0.53, 95% CI 0.32-0.88), not enough training or support (OR 0.44, 95% CI 0.26-0.74), accuracy concerns (OR 0.41, 95% CI 0.25-0.69), not being confident judging outputs (OR 0.24, 95% CI 0.14-0.43), and preferring human judgment (OR 0.24, 95% CI 0.14-0.40). Unclear or restrictive workplace policy, workplace norms discouraging use, client discomfort, and overreliance were not significantly associated with either outcome after correction.

**Table 5.** Univariable logistic regression analysis of endorsed concerns associated with daily administrative or clinician-support AI use and daily client-facing AI use.

Concern	Administrative OR^a^ (95% CI)	*P* value	Client-facing OR (95% CI)	*P* value
Privacy/security	0.95 (0.50-1.79)	.87	0.53 (0.32-0.88)	.03^b^
Workplace banned/unclear policy	1.55 (0.69-3.50)	.53	0.61 (0.34-1.07)	.13
Workplace norms discourage	1.33 (0.56-3.16)	.67	1.04 (0.55-1.96)	.91
Not enough training/support	1.32 (0.65-2.68)	.67	0.44 (0.26-0.74)	.005^b^
Accuracy	0.51 (0.26-0.98)	.17	0.41 (0.25-0.69)	.002^b^
Not confident judging outputs	1.12 (0.55-2.28)	.86	0.24 (0.14-0.43)	<.001^b^
Clients uncomfortable	2.02 (0.93-4.37)	.17	1.29 (0.75--2.22)	.39
Prefer human judgment	0.39 (0.21-0.73)	.03^b^	0.24 (0.14-0.40)	<.001^b^
Overreliance	0.56 (0.30-1.05)	.17	0.78 (0.47-1.30)	.39

a OR: odds ratio.

b Statistical significance at false discovery rate–adjusted *P*<.05.

#### Workplace Permissions for AI Use

**Figure 2.**
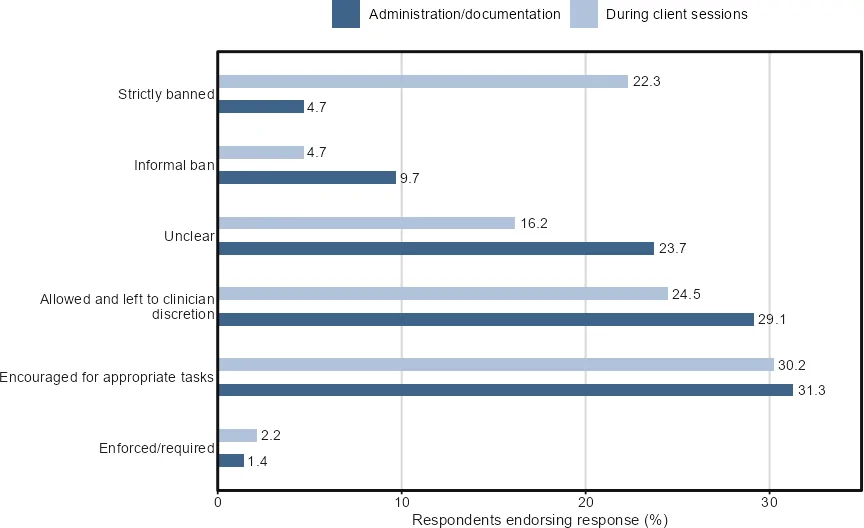
Workplace permissions for AI use in administrative/documentation tasks and during client sessions.

Workplace permissions for AI use in administrative or documentation tasks and during client sessions are shown in Figure 2. For administrative or documentation tasks, the most common responses were that AI was encouraged for appropriate tasks (87/278, 31.3%), AI was allowed at clinician discretion (81/278, 29.1%), or workplace rules were unclear (66/278, 23.7%). Permissions for use during client sessions were more mixed: 30.2% (84/278) reported that AI was encouraged for appropriate tasks, 24.5% (68/278) reported that it was allowed at clinician discretion, 22.3% (62/278) reported that it was strictly banned, and 16.2% (45/278) reported that workplace rules were unclear. Formal requirements were uncommon in both contexts (4/278, 1.4% for administrative or documentation tasks and 6/278, 2.2% for use during client sessions).

#### Multivariable Regression and Sensitivity Analysis

Multivariable logistic regression results are presented in Table 6. In adjusted cross-sectional analyses, speaking a language other than English at home was statistically associated with both daily AI use outcomes. Respondents who spoke a language other than English at home had higher odds of reporting daily administrative or clinician-support AI use (adjusted OR 2.62, 95% CI 1.54-4.53) and daily client-facing AI use (adjusted OR 2.36, 95% CI 1.36-4.16). Leave-one-task-out sensitivity analyses are presented in Tables S5 and S6 in Multimedia Appendix 1. The association between speaking a language other than English at home and daily client-facing AI use remained after separately excluding language translation (adjusted OR 3.05, 95% CI 1.73‐5.37), session transcription (adjusted OR 3.18, 95% CI 1.70‐5.95), and direct client chatting (adjusted OR 3.18, 95% CI 1.77‐5.71). The association with administrative or clinician-support use also remained after excluding research and information finding, personal emotional support, or administrative document preparation (adjusted OR range 1.93‐2.93), indicating that the primary associations were not attributable to any single high-frequency task. Sensitivity analyses using a weekly-or-more threshold are presented in Table S7 in Multimedia Appendix 1. The results broadly aligned with the daily-threshold models. Speaking a language other than English at home remained associated with weekly-or-more client-facing AI use (adjusted OR 4.42, 95% CI 2.39‐8.18). The association with weekly-or-more administrative or clinician-support use was in the same direction but was attenuated and narrowly exceeded the conventional significance threshold (adjusted OR 2.10, 95% CI 1.00‐4.42). The weekly administrative outcome was highly prevalent, with only 47 respondents classified as using AI less than weekly. Estimates from this model, particularly the profession estimates with wide CIs, should therefore be interpreted cautiously.

**Table 6.** Multivariable logistic regression models of factors associated with daily administrative or clinician-support AI use and daily client-facing AI use.

Predictor	Administrative aOR^a^ (95% CI)	*P* value	Client-facing aOR (95% CI)	*P* value
Age	1.00 (0.95-1.04)	.90	1.01 (0.96-1.06)	.69
Years practicing	1.02 (0.96-1.09)	.50	1.05 (0.98-1.12)	.17
Male (vs female)	1.44 (0.86-2.44)	.17	0.89 (0.51-1.54)	.68
Language other than English at home: yes (vs no)	2.62 (1.54-4.53)	<.001^b^	2.36 (1.36-4.16)	.003^b^
Master’s degree/doctorate (vs all others)	0.97 (0.56-1.66)	.90	1.12 (0.63-1.97)	.70
Counselor (vs psychologist)	0.82 (0.40-1.66)	.58	0.73 (0.34-1.52)	.40
Social worker (vs psychologist)	0.93 (0.40-2.12)	.86	0.82 (0.33-1.95)	.66
Occupational therapist (vs psychologist)	2.28 (0.97-5.47)	.06	1.65 (0.69-3.92)	.25
Psychiatrist (vs psychologist)	2.51 (0.99-6.62)	.06	1.24 (0.47-3.18)	.66
Other (vs psychologist)	0.97 (0.40-2.31)	.95	0.52 (0.18-1.36)	.20

a aOR: adjusted odds ratio.

b Statistical significance at *P*<.05.

### Integrated Findings

Across both phases, AI was positioned primarily as a clinician-support tool. Interview participants described using AI for documentation, research, planning, translation, and reducing cognitive load, while the survey showed that research and information finding (59/278, 21.2%), translation (51/278, 18.3%), and transcription (49/278, 17.6%) were the most common daily uses. AI was also rated most favorably for administrative documents, research, and transcription, while saving time and improving productivity were the most frequently endorsed reasons for use. Together, these findings suggest that perceived value was the greatest for workflow-support tasks rather than autonomous therapeutic delivery.

Both phases also identified privacy, accuracy, and human judgment as central concerns. Privacy and security concerns (175/278, 62.9%) and accuracy concerns (158/278, 56.8%) were the most frequently endorsed survey concerns, consistent with interview accounts concerning confidentiality, uncertain data handling, inaccurate outputs, and the need to review and edit AI-generated material. Across the 2 phases, perceived usefulness appeared the strongest when clinicians could review AI outputs and retain responsibility for judgment.

Governance findings were also consistent across phases. Interview participants described workplace bans, unclear policies, informal use, and unmet training needs, while survey respondents reported varied permissions, particularly during client sessions, where 22.3% (62/278) reported that AI was banned and 16.2% (45/278) reported unclear rules. Together, these findings indicate that AI uptake is occurring within an uneven governance environment rather than under consistent organizational guidance.

## Discussion

### Principal Findings

This study examined how general-purpose, off-the-shelf AI tools are being integrated into routine mental health practice, including their uses, perceived value, limitations, and workplace context. Overall, the findings indicate that AI is being adopted across both clinician-support and client-facing activities, but its clearest current role is as a clinician-supervised workflow support tool. Privacy, accuracy, human judgment, training, and uneven workplace governance remain important constraints. Speaking a language other than English at home was independently associated with daily AI use, whereas age, years practicing, gender, education, and profession were not associated with daily AI use.

The breadth of use observed in this study is consistent with emerging evidence that generative AI is being adopted across multiple clinical workflow functions [4,13,14], and this is extended by showing that clinicians perceived the greatest value in supervised workflow-support tasks rather than autonomous therapeutic delivery.

The absence of independent associations with age or years of practice suggests that uptake may not be confined to younger or less experienced clinicians. The relevant question may therefore be shifting from whether clinicians will adopt these tools to how their use can be governed, evaluated, and supported appropriately.

Implementation is also occurring through a changing ecosystem of general-purpose tools rather than a single standardized clinical platform. This makes regulation and evaluation difficult because clinicians and organizations may use different tools for similar tasks, while the capabilities, privacy arrangements, and outputs of those tools continue to change. Research and governance may therefore need to evaluate the categories of use and their associated risks rather than concentrating exclusively on individual platforms.

The association between speaking a language other than English at home and daily AI use warrants further investigation. Its persistence after excluding language translation from the client-facing outcome makes a translation-only explanation unlikely. AI may serve broader communication-support functions for some multilingual clinicians, but the broad language measure and cross-sectional design prevent this interpretation from being confirmed. Task-specific and longitudinal research is needed, particularly given emerging interest in AI-supported communication in non-English clinical settings [15].

The findings support viewing general-purpose AI as an augmentation tool rather than a substitute for clinical expertise. Clinician review, editing, and judgment remain important safeguards when outputs may be inaccurate, incomplete, or contextually inappropriate. Direct client-facing use requires particular attention because the survey did not establish what “chatting directly with a client” involved. This could include therapist-supervised assistance with low-risk asynchronous communication [16], AI-generated drafts subsequently reviewed by clinicians, or the use of AI-drafted replies to client messages [17,18]. Future research should distinguish these practices from autonomous interaction because they involve substantially different levels of clinical and ethical risk.

Participants also described AI as serving a personal support function outside formal sessions, with both clinicians and clients using it for reflection, validation, and coping. This suggests that AI may sometimes operate as an informal adjunct when human support is unavailable, including between appointments, late at night, or during periods of stress and burnout. This is particularly relevant given the high levels of burnout and workforce strain reported among mental health professionals, including psychiatrists [19] and psychologists [20], which can adversely affect the effectiveness of their psychotherapy delivery [21]. Previous studies of AI scribes have reported reductions in documentation burden, cognitive task load, and after-hours work, alongside improved attention during consultations [22-25]. This suggests that AI may be filling support gaps in an overstretched mental health system, functioning not only as a workflow aid but also as a resource that can make clinical work more psychologically manageable.

Privacy and security were the most frequently endorsed concerns, consistent with previous clinician research [4] and systematic review evidence [7]. Extending this literature, these concerns were associated with lower odds of daily client-facing AI use but not administrative or clinician-support AI use, while interview participants described clients declining AI-assisted processes. However, concurrent measurement precludes determining the direction of this association.

Our study extends this literature by showing how these concerns relate to routine practice. Interview participants described clients declining AI-assisted processes because of privacy concerns, while survey endorsement of privacy or security concerns was associated with lower odds of daily client-facing AI use but not administrative or clinician-support AI use. This task-specific pattern suggests that privacy concerns may be particularly salient when AI is used in direct client interactions or with sensitive client information, rather than reflecting a general objection to AI.

The prominence of privacy and security concerns has direct regulatory relevance. Processing identifiable client information through chatbots, AI scribes, transcription systems, or cloud services may engage the Privacy Act 1988 and Australian Privacy Principles [26,27]. These frameworks regulate the collection, use, disclosure, security, and cross-border handling of personal information, with health information receiving additional protection as sensitive information [26,27]. Australian guidance consequently emphasizes privacy impact assessment, data minimization, vendor scrutiny, staff training, human oversight, and avoiding sensitive information in publicly available generative AI tools [28].

Applicable requirements also vary by information type, jurisdiction, and practice setting. The My Health Records Act 2012 imposes additional access, security, auditing, and breach-response requirements when My Health Record information is involved [29]. Privacy and health-information laws also differ across states and territories and between public- and private-sector services [30,31]. Differences in organizational policies may therefore reflect uncertainty about how existing legal obligations should be translated into practical guidance concerning approved tools, consent, identifiable information, data storage, and review of AI-generated records.

Associations between endorsed concerns and daily AI use should be interpreted cautiously. Because concerns and use were measured concurrently, temporal ordering cannot be established. Respondents with less frequent AI use may have been more likely to endorse concerns, whereas greater familiarity among daily users may have influenced perceptions of risk. Conversely, pre-existing concerns may have discouraged use. These findings should therefore be interpreted as identifying potential barriers to adoption rather than evidence of particular concerns causing lower use. Longitudinal research is needed to distinguish among these explanations [32,33].

AI appeared to be used for both administrative and client-facing tasks even where workplace policy was unclear or restrictive, suggesting that uptake was not limited to settings with clear endorsement. This extends existing governance literature by showing that AI use may continue even when workplace policies are unclear or restrictive, supporting calls for clear organizational guidance, workforce training, and human oversight [7]. This implies that prohibition alone may be insufficient and that governance may be more effective when paired with practical guidance, education, and training on appropriate use. This interpretation is supported by the qualitative findings, in which many participants wanted more training but reported limited access to it. Organizations may therefore need to prioritize upskilling and clear guidance rather than relying primarily on enforcement [34,35].

Future uptake may be shaped by not only therapeutic value but also broader economic and system pressures. Clinicians may value AI most as a support tool that reduces burden without replacing relational work, whereas organizations and industry actors may be more strongly motivated by scalability, efficiency, and cost reduction. This creates a potential tension between what is clinically valued and what is economically incentivized. AI may therefore be adopted not only because it is considered therapeutically useful, but also because it is seen as a more scalable or less costly mode of support. It is important that this is carefully considered, given the variable impact it may have on clinician employment, as well as patient satisfaction and outcomes.

### Limitations

This study relied on convenience sampling and self-report data, which may have introduced selection and reporting biases. Clinicians with greater interest in AI, stronger views about AI, more experience using AI, or greater comfort with digital technologies may have been more likely to participate, particularly given recruitment through professional networks, social media, organizations, newsletters, and direct clinic contact.

Differences between the sample and national workforce profiles further limit representativeness. Consequently, the reported proportions describe the responding sample and should not be interpreted as prevalence estimates for the broader Australian mental health workforce. Compared with the national specialist mental health workforce, women were less represented in our sample (58.1%) than among psychologists (80.6%), mental health occupational therapists (85.0%), and accredited mental health social workers (83.7%), but more represented than among psychiatrists (43.2%) [36]. The sample also appeared relatively young, although comparisons were limited because national age profiles differed by profession and national datasets did not cover counselors or all other professions included in this study [36].

Some qualitative participants had prior professional relationships with members of the research team, which may have influenced their responses. Member checking was not undertaken, and thus, participants did not review transcripts or comment on the researchers’ interpretations. However, credibility was supported through independent coding by 2 researchers, consensus discussions, wider-team review of categories and themes, and the presentation of participant quotations.

The findings also represent a time-sensitive snapshot because AI tools, patterns of use, and workplace governance are changing rapidly. Furthermore, because the study was conducted in Australia, the findings may not generalize to service systems and regulatory contexts in other countries.

Some survey items may have captured heterogeneous practices. In particular, items, such as “chatting directly with a client” and “language translation,” may have been interpreted differently across respondents, and the survey could not fully capture variation within each task. Composite outcomes were used because the survey covered 12 distinct tasks and separate task-level regression models would have involved small numbers of daily users and potentially unstable estimates. However, this reduced task-level specificity, meaning the analyses could not determine which individual tasks primarily drove the reported associations.

The survey assessed AI use by task rather than by specific software or platform. We therefore could not determine which tools respondents used for each task, whether they used one multipurpose tool across several tasks, or whether they used multiple tools. This limits the interpretation of whether patterns of use and perceived performance differed between particular AI systems.

Finally, the survey was developed specifically for this study, using the qualitative findings, relevant literature, and author-team pretesting. It did not undergo external pilot testing or formal psychometric validation, limiting confidence in the reliability and validity of its items. Further validation in larger and more representative clinician samples is needed.

### Conclusions

The integration of general-purpose AI into mental health practice should be understood as a clinical governance challenge rather than solely a question of technology adoption. These tools may reduce administrative burden and support communication and clinical workflow, but their value depends on appropriate task selection, clinician oversight, protection of sensitive information, and preservation of professional accountability. Because adoption is occurring across a changing range of tools and workplace settings, governance and evaluation will need to be practical, task-specific, and adaptable. More representative, longitudinal, and task-level research is needed to determine how these technologies affect clinicians, therapeutic relationships, and patient care over time.

## Supplementary material

10.2196/101629 Multimedia Appendix 1 Supplementary information to support the study findings.

10.2196/101629 Checklist 1 COREQ qualitative checklist.

## Abbreviations

COREQ: Consolidated Criteria for Reporting Qualitative Research
LLM: large language model
OR: odds ratio
