# Supplementary material for: General-Purpose Artificial Intelligence Use in Routine Mental Health Practice Among Australian Clinicians: Mixed Methods Study
Source: J Med Internet Res. 2026 Aug 31;28:e101629. doi: 10.2196/101629 (PMC13528824; doi:10.2196/101629)
Supplement: Supplementary file 1 [file jmir-v28-e101629-s001.docx]

# **Supplement Appendix 1. Qualitative Interview Guide**

**Introduction and Verbal Consent**

Thank you for taking the time to participate in this study. Before we begin, I’d like to confirm that you’ve had a chance to review the Participant Information Sheet.

1. Have you read the information provided about this study?
2. Do you have any questions or concerns about the study that you’d like me to clarify before we begin?

*(Pause to allow participant to ask questions. Answer any clarifications needed.)*

Before proceeding, I need to confirm your consent:

- This study aims to explore how mental health professionals perceive and use large language models (LLMs) like ChatGPT in their clinical practice.
- Your participation is voluntary. You do not need to answer any questions you do not want to, and you may pause or stop the interview at any time.
- This interview will be audio-recorded for transcription and analysis. No personally identifiable information will be included in any reports or publications.
- If you have any concerns, you can contact the research team or UQ’s ethics office, as outlined in the Participant Information Sheet.

Do you consent to participating in this study?
Do you consent to this interview being audio-recorded?

*(Wait for participant’s verbal "yes" or "no" before proceeding.)*

## **Questions (Used as a Guide)**

**Demographic & Professional Background**

1. What is your current job title and role?
2. What type of workplace do you work in? (e.g., private practice, hospital, community clinic, telehealth, university setting)
3. What is your educational background and training in mental health?
4. How long have you been practicing therapy?
5. What populations do you primarily work with? (e.g., adults, children, specific mental health concerns, marginalized groups)
6. What therapy modality or modalities do you primarily work with? (e.g., CBT, ACT, MI, Psychodynamic, Schema therapy, Integrative, etc.)

**Therapists' Experiences Using LLMs in Their Practice**

1. Are you currently using large language models (LLMs) like ChatGPT in your clinical practice? If so, in what capacity?
2. **How do you personally integrate LLMs into your workflow? (e.g., note-taking, treatment planning, client education, administrative work, direct client interaction?)**
3. **Beyond clinical work, have you used LLMs for professional development? (e.g., staying updated on therapy techniques, writing reports, generating resources)**
4. **Have you experimented with LLMs outside of work for personal use, self-care, or productivity? If so, how?**

**Factors Influencing Openness or Reluctance Toward AI in Therapy**

1. **What factors have influenced your decision to use or avoid AI tools in your practice?**
2. **Do you feel LLMs can enhance therapeutic work, or do they risk oversimplifying clinical practice? Why?**
3. **Have you ever recommended or discouraged clients from using AI-based mental health tools? Why or why not?**
4. **What are your biggest ethical concerns when it comes to using AI in therapy? (e.g., confidentiality, therapeutic integrity, over-reliance, misinformation)**
5. **Do you feel that AI-based mental health tools could replace certain aspects of therapy, or do you view them as supplementary?**

**Perceived Accuracy and Reliability of LLMs**

1. How accurate do you believe LLMs are when providing psychological insights or guidance?
2. Would you trust AI-generated responses for assessing mental health conditions such as depression, anxiety, or ADHD? Why or why not?
3. Have you noticed instances where AI tools provided inaccurate, misleading, or potentially harmful advice? If so, what were the issues?
4. How do you currently verify or cross-check information provided by AI tools?

**Workplace Policies & AI Integration**

1. Does your workplace have any official policies or guidelines regarding the use of AI tools in clinical practice?
2. Have you received any training or guidance on AI use in therapy? If not, do you think such training should be required?
3. Are mental health workers in your organization allowed to use AI for clinical tasks such as note-taking, treatment planning, or client communication? If so, under what conditions?

**Final Confirmation & Wrap-Up:**

- **Where do you see it going in 5 years?**
- Is there anything else you’d like to add about your experience with AI in clinical practice?
- Do you have any questions for me before we conclude?

# **Supplement Figure 1. Self-reported reasons for using AI in therapeutic practice (multi-select)**

# **Supplement Figure 2. Endorsed concerns to using AI in therapeutic practice (multi-select)**

# **Supplement Appendix 2. COREQ Qualitative Checklist**

| **No.** | **Domain** | **Subdomain** | **Item** | **Guide questions/description** | **Reported in section** |
| --- | --- | --- | --- | --- | --- |
| 1 | Research team and reflexivity | Personal characteristics | Interviewer/facilitator | Which author/s conducted the interview or focus group? | Methods: Qualitative phase, Recruitment and data collection |
| 2 | Research team and reflexivity | Personal characteristics | Credentials | What were the researcher’s credentials? (e.g. PhD, MD) | Methods: Qualitative phase, Recruitment and data collection |
| 3 | Research team and reflexivity | Personal characteristics | Occupation | What was their occupation at the time of the study? | Methods: Qualitative phase, Recruitment and data collection |
| 4 | Research team and reflexivity | Personal characteristics | Gender | Was the researcher male or female? | Supplement C: BJ identified as male and TTY identified as female. |
| 5 | Research team and reflexivity | Personal characteristics | Experience and training | What experience or training did the researcher have? | Methods: Qualitative phase, Recruitment and data collection |
| 6 | Research team and reflexivity | Relationship with participants | Relationship established | Was a relationship established prior to study commencement? | Methods: Qualitative phase, Recruitment and data collection |
| 7 | Research team and reflexivity | Relationship with participants | Participant knowledge of the interviewer | What did the participants know about the researcher? (e.g. personal goals, reasons for doing the research) | Supplement C: Participants were informed about the purpose of the study, the research team’s interest in understanding AI use in therapeutic practice, and the voluntary nature of participation through the participant information and consent materials. |
| 8 | Research team and reflexivity | Relationship with participants | Interviewer characteristics | What characteristics were reported about the interviewer/facilitator? (e.g. bias, assumptions, reasons and interests in the research topic) | Methods: Qualitative phase, Recruitment and data collection; Qualitative analysis and survey development |
| 9 | Study design | Theoretical framework | Methodological orientation and theory | What methodological orientation was stated to underpin the study? (e.g. grounded theory, discourse analysis, ethnography, phenomenology, content analysis) | Methods: Qualitative phase, Qualitative analysis and survey development |
| 10 | Study design | Participant selection | Sampling | How were participants selected? (e.g. purposive, convenience, consecutive, snowball) | Methods: Qualitative phase, Recruitment and data collection |
| 11 | Study design | Participant selection | Method of approach | How were participants approached? (e.g. face-to-face, telephone, mail, email) | Supplement C: Participants were approached through LinkedIn messages sent via the research team’s professional networks. |
| 12 | Study design | Participant selection | Sample size | How many participants were in the study? | Methods: Qualitative phase, Recruitment and data collection; Results: Qualitative phase |
| 13 | Study design | Participant selection | Non-participation | How many people refused to participate or dropped out? Reasons? | Supplement C: Some individuals who expressed interest were unable to participate because of scheduling constraints. The number of non-participants was not systematically recorded. No participants dropped out after interview participation. |
| 14 | Study design | Setting | Setting of data collection | Where was the data collected? (e.g. home, clinic, workplace) | Methods: Qualitative phase, Recruitment and data collection |
| 15 | Study design | Setting | Presence of non-participants | Was anyone else present besides the participants and researchers? | Supplement C: Only the participant and interviewing researcher was present. |
| 16 | Study design | Setting | Description of sample | What are the important characteristics of the sample? (e.g. demographic data, date) | Results: Qualitative phase; Table 2 |
| 17 | Study design | Data collection | Interview guide | Were questions, prompts, or guides provided by the authors? Was it pilot tested? | Methods: Qualitative phase, Recruitment and data collection; Supplement A |
| 18 | Study design | Data collection | Repeat interviews | Were repeat interviews carried out? If yes, how many? | Supplement C: No repeat interviews were carried out |
| 19 | Study design | Data collection | Audio/visual recording | Did the research use audio or visual recording to collect the data? | Supplement C: The interviewers recorded via Zoom. |
| 20 | Study design | Data collection | Field notes | Were field notes made during and/or after the interview or focus group? | Supplement C: Field notes were not made during interview |
| 21 | Study design | Data collection | Duration | What was the duration of the interviews or focus group? | Supplement C: Interviews ran between 27 to 58 minutes, with an average length of 35 minutes. |
| 22 | Study design | Data collection | Data saturation | Was data saturation discussed? | Methods: Qualitative phase, Recruitment and data collection |
| 23 | Study design | Data collection | Transcripts returned | Were transcripts returned to participants for comment and/or correction? | Methods: Qualitative phase, Qualitative analysis and survey development |
| 24 | Analysis and findings | Data analysis | Number of data coders | How many data coders coded the data? | Methods: Qualitative phase, Qualitative analysis and survey development |
| 25 | Analysis and findings | Data analysis | Description of the coding tree | Did authors provide a description of the coding tree? | Table 1 |
| 26 | Analysis and findings | Data analysis | Derivation of themes | Were themes identified in advance or derived from the data? | Methods: Qualitative phase, Qualitative analysis and survey development |
| 27 | Analysis and findings | Data analysis | Software | What software, if applicable, was used to manage the data? | Methods: Qualitative phase, Qualitative analysis and survey development |
| 28 | Analysis and findings | Data analysis | Participant checking | Did participants provide feedback on the findings? | Methods: Qualitative phase, Qualitative analysis and survey development |
| 29 | Analysis and findings | Reporting | Quotations presented | Were participant quotations presented to illustrate the themes/findings? Was each quotation identified? (e.g. participant number) | Results: Qualitative phase |
| 30 | Analysis and findings | Reporting | Data and findings consistent | Was there consistency between the data presented and the findings? | Results: Qualitative phase; Table 1 |
| 31 | Analysis and findings | Reporting | Clarity of major themes | Were major themes clearly presented in the findings? | Results: Qualitative phase; Table 1 |
| 32 | Analysis and findings | Reporting | Clarity of minor themes | Is there a description of diverse cases or discussion of minor themes? | Results: Qualitative phase; Table 1 |

# **Supplement Table 1. Developmental joint display linking qualitative interview findings to quantitative survey items**

| **Qualitative theme and subtheme** | **Interview-derived findings** | **Exact quantitative survey item(s) and nature of integration** |
| --- | --- | --- |
| Theme 1: Current functions and perceived effectiveness — 1.1 In-session use | AI-assisted transcription, note-taking, and case-note generation; reduced cognitive load; improved focus and therapeutic engagement during sessions; translation when an interpreter was unavailable | Q1: “In the past 8 weeks, how often did you use AI for each of the following tasks?” Items: “To transcribe sessions or take notes” and “For language translation.” Respondents who had used these tasks also received Q2: “How effective is AI for the following tasks in your personal experience?” and Q3: “Compared with a human for this task, AI is…” Q4: “Why do you use AI?” Items: “AI lets me stay more present in session by reducing admin and note-taking,” “AI reduces cognitive load and gives me more recovery time between sessions,” and “AI helps me bridge language barriers (e.g., translation or simplifying English).” These items directly operationalised the in-session tasks and benefits described in the interviews. |
| Theme 1: Current functions and perceived effectiveness — 1.2 Out-of-session use | Drafting letters, reports, emails, and referrals; treatment planning and clinical brainstorming; development of psychoeducation materials; research and unfamiliar-topic learning; provisional diagnostic prompting | Q1: “To prepare administrative documents (emails, referrals, etc.),” “For treatment planning (interventions, session plans),” “For client education (handouts, worksheets, resources),” “For research and information finding,” “For informal supervision or guidance (advice on cases),” and “For formal diagnosis.” Respondents who reported using these tasks also received the corresponding Q2 effectiveness and Q3 human-comparison items. Q4: “AI saves me time and allows me to be more productive,” “AI improves the quality of my work and client care,” and “AI expands my professional skillset.” These items directly captured the administrative, planning, education, research, and guidance functions described by participants. The formal-diagnosis item extended the narrower interview finding concerning provisional diagnostic prompting. |
| Theme 1: Current functions and perceived effectiveness — 1.3 Direct psychological support | Clinicians used AI for emotional validation and reflection; clients used AI for coping support between appointments; participants valued its immediate availability | Q1: “To chat directly with a client (AI interacts with client for therapeutic support)” and “For your own emotional support (self-care, encouragement, comfort).” Respondents who had used these tasks also received the corresponding Q2 effectiveness and Q3 human-comparison items. These items directly assessed the client-support and clinician-support functions identified in the interviews. The perceived benefit of continuous or 24-hour availability was not measured as a separate survey item. |
| Theme 2: Present limitations and reasons for non-use — 2.1 Accuracy and reliability concerns | Hallucinated references; misleading, incomplete, or overly general information; inaccurate transcription or omission of session details; need to check and verify outputs | Q5: “Does any of the following stop you or make you reluctant to use AI?” Items: “I am concerned about AI’s accuracy” and “I am not confident in my ability to judge AI outputs safely.” Q2 and Q3 also assessed task-specific effectiveness and perceived performance relative to humans. The cocnern items directly operationalised participants’ accuracy and verification concerns, while Q2 and Q3 assessed whether these concerns varied across individual AI-use tasks. |
| Theme 2: Present limitations and reasons for non-use — 2.2 Privacy, security, and client acceptability | Confidentiality concerns; uncertainty about the storage, ownership, and use of information entered into AI systems; client discomfort and refusal to consent | Q5: “I have concerns about privacy and security” and “My clients are uncomfortable with or skeptical about AI.” These items directly assessed the privacy and client-acceptability concerns reported in the interviews. Concerns about data storage, ownership, and secondary use were represented within the broader privacy and security item rather than measured separately. |
| Theme 2: Present limitations and reasons for non-use — 2.3 The human touch | AI lacked genuine attunement, emotional understanding, and sensitivity to nonverbal cues; participants emphasised the importance of human judgement and the therapeutic relationship | Q3: “Compared with a human for this task, AI is…” Respondents rated AI from “Much worse” to “Much better” for each task they had used. Q5: “I prefer human judgment or supervision over AI assistance” and “I am concerned about becoming over-reliant on AI.” These items assessed the perceived comparative value of human input and concerns about substituting AI for clinician judgement. Specific relational qualities, including genuine empathy, attunement, and interpretation of body language, were not assessed as separate survey items. |
| Theme 3: Integration into practice — 3.1 Future projections of AI in mental health therapy | AI was generally viewed as a supplement rather than a replacement for clinicians; some participants anticipated replacement of discrete or highly structured tasks; one participant raised concern about adoption being driven by economic or corporate pressure | Q3: “Compared with a human for this task, AI is…” Q5: “I prefer human judgment or supervision over AI assistance” and “I am concerned about becoming over-reliant on AI.” These items indirectly assessed whether AI was perceived as an adjunct or potential substitute for human-delivered work. The possibility that adoption could be driven by corporate or economic pressure was not directly assessed in the survey. |
| Theme 3: Integration into practice — 3.2 Influence of workplace policy and training | Uneven workplace governance; explicit bans or replacement of one AI platform with another; uncertainty about permissible use; need for practical training and guidance | Q4: “AI use aligns with my workplace norms (it’s encouraged and commonly used by colleagues).” Q5: “My workplace has banned AI use or has unclear policies about it,” “Workplace norms discourage AI use (it isn’t used among colleagues),” and “I have not received enough training or support to use AI effectively.” Q6: “For the statements below, indicate the option that best describes your workplace:” Items: “My workplace allows AI tools for administration or documentation tasks (e.g., writing case notes, letters, scheduling, formatting)” and “My workplace allows me to use AI tools during client sessions (e.g., to transcribe sessions, create case notes).” Q7: “My workplace has clear written AI policies or guidelines.” These items directly operationalised organisational permission, restrictions, workplace norms, written governance, and training needs identified in the interviews. |
| Items added during literature review and author-team refinement | These items did not have a close or sufficiently specific corresponding interview category | Q1: “To practice skills (role-playing, hypothetical cases)” and “For screening or triage questionnaires (not formal diagnosis).” Q4: “AI lowers my costs for training, supervision, or administration.” Q5: “I do not see a clear benefit of AI for my role” and “I have no concerns about using AI.” These items were added to broaden content coverage, capture contrasting perspectives, or provide complete response options rather than being directly derived from an individual qualitative category. |

# **Supplement Table 2. Bivariate comparisons of participant characteristics by daily administrative or clinician-support and client-facing AI use**

| Characteristic | Administrative daily use, n/N (%) | p | Client-facing daily use, n/N (%) | p |
| --- | --- | --- | --- | --- |
| Gender |  | .196 |  | .593 |
| Female | 64/160 (40.0) |  | 55/160 (34.4) |  |
| Male | 55/115 (47.8) |  | 36/115 (31.3) |  |
| Language other than English at home |  | .001* |  | .005* |
| No | 55/157 (35.0) |  | 41/157 (26.1) |  |
| Yes | 64/118 (54.2) |  | 50/118 (42.4) |  |
| Master’s degree or doctorate |  | .450 |  | .101 |
| No | 51/125 (40.8) |  | 35/125 (28.0) |  |
| Yes | 68/150 (45.3) |  | 56/150 (37.3) |  |
| Profession |  | .094 |  | .446 |
| Psychologist | 34/84 (40.5) |  | 29/84 (34.5) |  |
| Counsellor | 24/67 (35.8) |  | 20/67 (29.9) |  |
| Social worker | 15/36 (41.7) |  | 11/36 (30.6) |  |
| Occupational therapist | 18/31 (58.1) |  | 13/31 (41.9) |  |
| Psychiatrist | 16/25 (64.0) |  | 11/25 (44.0) |  |
| Other | 12/32 (37.5) |  | 7/32 (21.9) |  |

# **Supplement Table 3. Daily artificial intelligence use by individual task in the past 8 weeks**

| **Task** | **Daily use, n (%)** |
| --- | --- |
| Chat directly with a client | 26 (9.2%) |
| Transcribe sessions | 49 (17.4%) |
| Prepare administrative documents | 36 (12.8%) |
| Treatment planning | 18 (6.4%) |
| Client education | 26 (9.2%) |
| Research and information finding | 59 (20.9%) |
| Practice skills | 24 (8.5%) |
| Language translation | 51 (18.1%) |
| Screening or triage questionnaires | 19 (6.7%) |
| Formal diagnosis | 14 (5.0%) |
| Informal supervision or guidance | 19 (6.7%) |
| Clinician’s own emotional support | 45 (16.0%) |

# **Supplementary Table 4. Perceived performance of artificial intelligence relative to humans by task**

| **Task** | **Valid n** | **Much worse, n (%)** | **Slightly worse, n (%)** | **About the same, n (%)** | **Slightly better, n (%)** | **Much better, n (%)** |
| --- | --- | --- | --- | --- | --- | --- |
| Chat directly with a client | 142 | 15 (10.6%) | 37 (26.1%) | 29 (20.4%) | 37 (26.1%) | 24 (16.9%) |
| Transcribe sessions or take notes | 207 | 4 (1.9%) | 13 (6.3%) | 50 (24.2%) | 88 (42.5%) | 52 (25.1%) |
| Prepare administrative documents | 253 | 4 (1.6%) | 18 (7.1%) | 49 (19.4%) | 109 (43.1%) | 73 (28.9%) |
| Treatment planning | 204 | 13 (6.4%) | 52 (25.5%) | 59 (28.9%) | 59 (28.9%) | 21 (10.3%) |
| Client education | 237 | 7 (3.0%) | 25 (10.5%) | 66 (27.8%) | 90 (38.0%) | 49 (20.7%) |
| Research and information finding | 259 | 9 (3.5%) | 31 (12.0%) | 41 (15.8%) | 92 (35.5%) | 86 (33.2%) |
| Practice skills | 186 | 13 (7.0%) | 47 (25.3%) | 47 (25.3%) | 57 (30.6%) | 22 (11.8%) |
| Language translation | 207 | 8 (3.9%) | 20 (9.7%) | 49 (23.7%) | 75 (36.2%) | 55 (26.6%) |
| Screening or triage questionnaires | 168 | 5 (3.0%) | 35 (20.8%) | 46 (27.4%) | 56 (33.3%) | 26 (15.5%) |
| Formal diagnosis | 106 | 4 (3.8%) | 16 (15.1%) | 38 (35.8%) | 29 (27.4%) | 19 (17.9%) |
| Informal supervision or guidance | 163 | 4 (2.5%) | 43 (26.4%) | 42 (25.8%) | 56 (34.4%) | 18 (11.0%) |
| Clinician’s own emotional support | 190 | 11 (5.8%) | 40 (21.1%) | 35 (18.4%) | 60 (31.6%) | 44 (23.2%) |

# **Supplementary Table 5A. Leave-one-task-out sensitivity analyses for daily administrative or clinician-support AI use**

| **Predictor** | **Excluding translation AOR (95% CI)** | **p** | **Excluding transcription AOR (95% CI)** | **p** | **Excluding direct client chatting AOR (95% CI)** | **p** |
| --- | --- | --- | --- | --- | --- | --- |
| Age | 1.04 (0.99 to 1.09) | .111 | 0.99 (0.94 to 1.05) | .781 | 1.00 (0.96 to 1.05) | .867 |
| Years practised | 0.96 (0.90 to 1.03) | .273 | 1.04 (0.96 to 1.12) | .325 | 1.07 (1.00 to 1.14) | .057 |
| Male (vs female) | 1.58 (0.91 to 2.75) | .104 | 0.88 (0.47 to 1.63) | .682 | 0.90 (0.51 to 1.60) | .721 |
| Language other than English at home: yes (vs no) | 3.05 (1.73 to 5.37) | <.001* | 3.18 (1.70 to 5.95) | <.001* | 3.18 (1.77 to 5.71) | <.001* |
| Master’s degree or doctorate (vs all other) | 1.16 (0.66 to 2.05) | .603 | 1.49 (0.78 to 2.83) | .228 | 1.26 (0.70 to 2.29) | .437 |
| Counsellor (vs psychologist) | 0.38 (0.17 to 0.82) | .014* | 0.89 (0.39 to 2.05) | .785 | 0.82 (0.38 to 1.77) | .616 |
| Social worker (vs psychologist) | 1.09 (0.47 to 2.55) | .834 | 0.91 (0.34 to 2.43) | .850 | 0.67 (0.26 to 1.72) | .408 |
| Occupational therapist (vs psychologist) | 3.11 (1.20 to 8.05) | .019* | 2.36 (0.92 to 6.09) | .076 | 1.37 (0.55 to 3.43) | .500 |
| Psychiatrist (vs psychologist) | 1.12 (0.43 to 2.91) | .810 | 1.69 (0.59 to 4.84) | .330 | 1.05 (0.38 to 2.89) | .931 |
| Other profession (vs psychologist) | 0.59 (0.23 to 1.49) | .265 | 0.84 (0.29 to 2.48) | .757 | 0.60 (0.22 to 1.65) | .323 |

# **Supplement Table 5B. Leave-one-task-out sensitivity analyses for daily administrative or clinician-support AI use cont.**

| **Predictor** | **Excluding research AOR (95% CI)** | **p** | **Excluding emotional support AOR (95% CI)** | **p** | **Excluding administrative documents AOR (95% CI)** | **p** |
| --- | --- | --- | --- | --- | --- | --- |
| Age | 1.01 (0.97 to 1.06) | .681 | 0.99 (0.95 to 1.03) | .593 | 1.00 (0.95 to 1.04) | .840 |
| Years practised | 1.00 (0.94 to 1.07) | .989 | 1.01 (0.95 to 1.08) | .669 | 1.03 (0.97 to 1.10) | .379 |
| Male (vs female) | 1.30 (0.75 to 2.24) | .347 | 1.29 (0.76 to 2.17) | .343 | 1.68 (0.99 to 2.86) | .056 |
| Language other than English at home: yes (vs no) | 2.93 (1.67 to 5.12) | <.001* | 1.93 (1.14 to 3.29) | .015* | 2.38 (1.37 to 4.14) | .002* |
| Master’s degree or doctorate (vs all other) | 1.11 (0.63 to 1.94) | .723 | 1.24 (0.72 to 2.12) | .441 | 0.81 (0.47 to 1.41) | .462 |
| Counsellor (vs psychologist) | 0.91 (0.44 to 1.92) | .814 | 0.84 (0.41 to 1.71) | .632 | 0.73 (0.35 to 1.52) | .404 |
| Social worker (vs psychologist) | 1.05 (0.45 to 2.49) | .904 | 0.96 (0.42 to 2.19) | .922 | 0.95 (0.41 to 2.20) | .913 |
| Occupational therapist (vs psychologist) | 2.59 (1.08 to 6.25) | .034* | 1.88 (0.81 to 4.41) | .144 | 2.43 (1.02 to 5.79) | .045* |
| Psychiatrist (vs psychologist) | 3.69 (1.40 to 9.73) | .008* | 1.39 (0.55 to 3.54) | .491 | 3.30 (1.25 to 8.72) | .016* |
| Other profession (vs psychologist) | 1.04 (0.41 to 2.61) | .936 | 1.10 (0.46 to 2.62) | .824 | 1.15 (0.48 to 2.75) | .758 |

# **Supplementary Table 6. Multivariable logistic regression models of factors associated with weekly-or-more administrative or clinician-support AI use and weekly-or-more client-facing AI use**

| **Predictor** | **Administrative AOR (95% CI)** | **P value** | **Client-facing AOR (95% CI)** | **P value** |
| --- | --- | --- | --- | --- |
| Age | 0.99 (0.94 to 1.04) | .650 | 1.03 (0.98 to 1.07) | .267 |
| Years practised | 1.02 (0.94 to 1.09) | .667 | 1.02 (0.96 to 1.09) | .483 |
| Male (vs female) | 1.92 (0.93 to 3.94) | .077 | 1.05 (0.60 to 1.85) | .857 |
| Language other than English at home: yes (vs no) | 2.10 (1.00 to 4.42) | .050 | 4.42 (2.39 to 8.18) | <.001* |
| Master’s degree or doctorate (vs all other) | 1.12 (0.55 to 2.29) | .754 | 1.09 (0.61 to 1.93) | .778 |
| Counsellor (vs psychologist) | 1.01 (0.41 to 2.49) | .991 | 0.49 (0.23 to 1.04) | .063 |
| Social worker (vs psychologist) | 0.59 (0.22 to 1.56) | .284 | 1.69 (0.66 to 4.32) | .277 |
| Occupational therapist (vs psychologist) | 6.87 (0.86 to 55.08) | .070 | 2.40 (0.88 to 6.53) | .087 |
| Psychiatrist (vs psychologist) | 5.81 (0.71 to 47.47) | .100 | 1.88 (0.64 to 5.54) | .250 |
| Other profession (vs psychologist) | 0.76 (0.28 to 2.07) | .588 | 0.48 (0.19 to 1.18) | .109 |
