# Supplementary material for: General-Purpose Artificial Intelligence Use in Routine Mental Health Practice Among Australian Clinicians: Mixed Methods Study
Source: J Med Internet Res. 2026 Aug 31;28:e101629. doi: 10.2196/101629 (PMC13528824; doi:10.2196/101629)
Supplement: Supplementary file 2 [file jmir-v28-e101629-s002.docx]

# **COREQ Qualitative Checklist**

| **No.** | **Domain** | **Subdomain** | **Item** | **Guide questions/description** | **Reported in section** |
| --- | --- | --- | --- | --- | --- |
| 1 | Research team and reflexivity | Personal characteristics | Interviewer/facilitator | Which author/s conducted the interview or focus group? | Methods: Qualitative phase, Recruitment and data collection |
| 2 | Research team and reflexivity | Personal characteristics | Credentials | What were the researcher’s credentials? (e.g. PhD, MD) | Methods: Qualitative phase, Recruitment and data collection |
| 3 | Research team and reflexivity | Personal characteristics | Occupation | What was their occupation at the time of the study? | Methods: Qualitative phase, Recruitment and data collection |
| 4 | Research team and reflexivity | Personal characteristics | Gender | Was the researcher male or female? | Supplement C: BJ identified as male and TTY identified as female. |
| 5 | Research team and reflexivity | Personal characteristics | Experience and training | What experience or training did the researcher have? | Methods: Qualitative phase, Recruitment and data collection |
| 6 | Research team and reflexivity | Relationship with participants | Relationship established | Was a relationship established prior to study commencement? | Methods: Qualitative phase, Recruitment and data collection |
| 7 | Research team and reflexivity | Relationship with participants | Participant knowledge of the interviewer | What did the participants know about the researcher? (e.g. personal goals, reasons for doing the research) | Supplement C: Participants were informed about the purpose of the study, the research team’s interest in understanding AI use in therapeutic practice, and the voluntary nature of participation through the participant information and consent materials. |
| 8 | Research team and reflexivity | Relationship with participants | Interviewer characteristics | What characteristics were reported about the interviewer/facilitator? (e.g. bias, assumptions, reasons and interests in the research topic) | Methods: Qualitative phase, Recruitment and data collection; Qualitative analysis and survey development |
| 9 | Study design | Theoretical framework | Methodological orientation and theory | What methodological orientation was stated to underpin the study? (e.g. grounded theory, discourse analysis, ethnography, phenomenology, content analysis) | Methods: Qualitative phase, Qualitative analysis and survey development |
| 10 | Study design | Participant selection | Sampling | How were participants selected? (e.g. purposive, convenience, consecutive, snowball) | Methods: Qualitative phase, Recruitment and data collection |
| 11 | Study design | Participant selection | Method of approach | How were participants approached? (e.g. face-to-face, telephone, mail, email) | Supplement C: Participants were approached through LinkedIn messages sent via the research team’s professional networks. |
| 12 | Study design | Participant selection | Sample size | How many participants were in the study? | Methods: Qualitative phase, Recruitment and data collection; Results: Qualitative phase |
| 13 | Study design | Participant selection | Non-participation | How many people refused to participate or dropped out? Reasons? | Supplement C: Some individuals who expressed interest were unable to participate because of scheduling constraints. The number of non-participants was not systematically recorded. No participants dropped out after interview participation. |
| 14 | Study design | Setting | Setting of data collection | Where was the data collected? (e.g. home, clinic, workplace) | Methods: Qualitative phase, Recruitment and data collection |
| 15 | Study design | Setting | Presence of non-participants | Was anyone else present besides the participants and researchers? | Supplement C: Only the participant and interviewing researcher was present. |
| 16 | Study design | Setting | Description of sample | What are the important characteristics of the sample? (e.g. demographic data, date) | Results: Qualitative phase; Table 2 |
| 17 | Study design | Data collection | Interview guide | Were questions, prompts, or guides provided by the authors? Was it pilot tested? | Methods: Qualitative phase, Recruitment and data collection; Supplement A |
| 18 | Study design | Data collection | Repeat interviews | Were repeat interviews carried out? If yes, how many? | Supplement C: No repeat interviews were carried out |
| 19 | Study design | Data collection | Audio/visual recording | Did the research use audio or visual recording to collect the data? | Supplement C: The interviewers recorded via Zoom. |
| 20 | Study design | Data collection | Field notes | Were field notes made during and/or after the interview or focus group? | Supplement C: Field notes were not made during interview |
| 21 | Study design | Data collection | Duration | What was the duration of the interviews or focus group? | Supplement C: Interviews ran between 27 to 58 minutes, with an average length of 35 minutes. |
| 22 | Study design | Data collection | Data saturation | Was data saturation discussed? | Methods: Qualitative phase, Recruitment and data collection |
| 23 | Study design | Data collection | Transcripts returned | Were transcripts returned to participants for comment and/or correction? | Methods: Qualitative phase, Qualitative analysis and survey development |
| 24 | Analysis and findings | Data analysis | Number of data coders | How many data coders coded the data? | Methods: Qualitative phase, Qualitative analysis and survey development |
| 25 | Analysis and findings | Data analysis | Description of the coding tree | Did authors provide a description of the coding tree? | Table 1 |
| 26 | Analysis and findings | Data analysis | Derivation of themes | Were themes identified in advance or derived from the data? | Methods: Qualitative phase, Qualitative analysis and survey development |
| 27 | Analysis and findings | Data analysis | Software | What software, if applicable, was used to manage the data? | Methods: Qualitative phase, Qualitative analysis and survey development |
| 28 | Analysis and findings | Data analysis | Participant checking | Did participants provide feedback on the findings? | Methods: Qualitative phase, Qualitative analysis and survey development |
| 29 | Analysis and findings | Reporting | Quotations presented | Were participant quotations presented to illustrate the themes/findings? Was each quotation identified? (e.g. participant number) | Results: Qualitative phase |
| 30 | Analysis and findings | Reporting | Data and findings consistent | Was there consistency between the data presented and the findings? | Results: Qualitative phase; Table 1 |
| 31 | Analysis and findings | Reporting | Clarity of major themes | Were major themes clearly presented in the findings? | Results: Qualitative phase; Table 1 |
| 32 | Analysis and findings | Reporting | Clarity of minor themes | Is there a description of diverse cases or discussion of minor themes? | Results: Qualitative phase; Table 1 |
